# TIAM1 variants improve clinical outcome in neuroblastoma

**DOI:** 10.18632/oncotarget.16787

**Published:** 2017-04-03

**Authors:** Elena Sanmartín, Yania Yáñez, Victoria Fornés-Ferrer, José L. Zugaza, Adela Cañete, Victoria Castel, Jaime Font de Mora

**Affiliations:** ^1^ Laboratory of Cellular and Molecular Biology, Instituto de Investigación Sanitaria La Fe, Valencia, Spain; ^2^ Pediatric Oncology Unit, Hospital Universitario y Politécnico La Fe, València, Spain; ^3^ Precision Oncology Unit, Instituto de Investigación Sanitaria La Fe, Valencia, Spain; ^4^ Biostatistics Unit, Instituto de Investigación Sanitaria La Fe, Valencia, Spain; ^5^ Department of Genetics, Physical Anthropology and Animal Physiology, University of the Basque Country, Leioa, Spain; ^6^ Achucarro Basque Center for Neuroscience, Bizkaia Science and Technology Park, Zamudio, Spain; ^7^ IKERBASQUE, Basque Foundation for Science, Bilbao, Spain

**Keywords:** neuroblastoma, TIAM1, signaling networks, next generation sequencing

## Abstract

Identification of tumor driver mutations is crucial for improving clinical outcome using a personalized approach to the treatment of cancer. Neuroblastoma is a tumor of the peripheral sympathetic nervous system for which only a few driver alterations have been described including *MYCN* amplification and *ALK* mutations. We assessed 106 primary neuroblastoma tumors by next generation sequencing using a customized amplicon-based gene panel. Our results reveal that genetic variants in *TIAM1* gene associate with better clinical outcome, suggesting a role for these *TIAM1* variants in preventing progression of this disease. The detected variants are located within the different domains of TIAM1 that signal to the upstream regulator RAS and downstream effector molecules MYC and RAC, which are all implicated in neuroblastoma etiology and progression. Clinical outcome was improved in tumors where a *TIAM1* variant was present concomitantly with either *ALK* mutation or *MYCN* amplification. Given the function of these signaling molecules in cell survival, proliferation, differentiation and neurite outgrowth, our data suggest that the *TIAM1*-mediated network is essential to neuroblastoma and thus, inhibiting *TIAM1* reflects a rational strategy for improving therapy efficacy in neuroblastoma.

## INTRODUCTION

TIAM1 is a molecular conduit to a complex network of signaling pathways that control cell-fate decisions. TIAM1 N-terminal region binds to MYC box II, a highly conserved domain among MYC proteins, to inhibit MYC-mediated apoptosis [1]. This region also functions as an auto-inhibitory loop which can be released upon phosphorylation by PKCs [2, 3]. In addition to MYC signaling, TIAM1 can also associate with activated RAS oncoprotein [4]. TIAM1-deficient mice are resistant to the development of RAS-induced skin tumors, revealing that TIAM1 is a critical regulator of RAS-induced tumor formation [5]. Closer to its N-terminus, the tyrosine 384 is subjected to SRC-dependent phosphorylation, which in turn recruits GRB2/SOS complex and leads to RAS/MAPK activation [6]. Therefore, TIAM1 has a dual function, signaling as an effector or as an activator molecule for RAS.

TIAM1 also activates RAC to stimulate membrane ruffling, cell migration and invasion [7]. RAC activation is catalyzed by TIAM1 Dbl homology domain (DH) that works in tandem with a pleckstrin homology domain (PH) [8, 9]. TIAM1 has an additional PH domain closer to the N-terminal region which determines the activation of distinct RAS-mediated signaling pathways such as c-Jun NH2-terminal kinase (JNK) [10]. Altogether, cellular and molecular data reveal TIAM1 as a molecular conduit to MYC, RAS and RAC signaling and integrates these three pathways in a network to orchestrate pleiotropic responses in the cell.

Cellular polarity in neuroblastoma cells is controlled by TIAM1-dependent RAC activation mediated by the polarity complex PAR-6-PAR-3 [11]. Nonetheless, the role of TIAM1 in neuroblastoma cells has been mostly focused in neuritogenesis. TIAM1 is a specific regulator of RAC during neuritogenesis [12] and is required for ephrin-B1 and EphA2 signaling in neurite outgrowth [13]. More recently, a whole-genome sequence analysis of 87 neuroblastomas showed three mutations in TIAM1 as well as other mutations in regulators of the Rac/Rho pathway, suggesting that defects in neuritogenesis may contribute to neuroblastoma [14].

In the present study, we screened primary neuroblastomas by next generation sequencing and our results reveal that *TIAM1* variants in the domains signaling to MYC, RAS and RAC significantly predict better prognosis in neuroblastoma patients. Moreover, three high-risk patients (age >1.5 years) with *MYCN* amplification also had a *TIAM1* variant but did not relapse. Notably, two of these patients are already free of disease. These novel observations implicate TIAM1 as a key signaling molecule in neuroblastoma and as a potential target for therapeutic intervention.

## RESULTS

### Technical characteristics of the NB-panel

To identify altered druggable targets and outcome predictors in neuroblastoma, we analyzed a collection of 106 primary tumors by next generation sequencing (NGS). A customized amplicon-based gene panel (NB-panel) containing 26 genes with implications in neuroblastoma or other cancers was designed and implemented (Table 1, see Supplementary Table 1 for genomic coordinates). Mean values and ranges of relevant technical parameters are shown in Table 2. Interestingly, many of the *MYCN* amplified cases displayed a rather low uniformity (less than 50%), with a strong deviation of the readings towards *MYCN* amplicons. This sequestering of the readings always coincided with *MYCN* amplification, suggesting an additional approach to predict amplification status by NGS. However, since some *MYCN* amplified cases displayed a reasonably high uniformity and equal distribution of the readings throughout the amplicons of the panel, this approach is not fully reliable as a predictor as it may fail to detect some positives. The sequestering of the readings towards *MYCN* sequencing may rely on other factors such as the percentage of neuroblasts in the sample or the ratio of *MYCN* amplification.

**Table 1:** Genes and covered exons included in the customized NB-panel

Gene	Locus	Ref Seq	Total exons	Covered exons
*ABL1*	9q	NM_007313.2	11	6
*AKT1*	14q	NM_001014431.1	14	1, 5-10
*ALK*	2p	NM_004304.4	29	20-29
*ARID1A*	1p	NM_006015.4	20	1-20
*ATM*	11q	NM_000051.3	63	9-12, 16-19, 21, 25-29, 31, 32, 37, 39-46, 54, 55, 58, 61-63
*ATRX*	Xq	NM_000489.4	35	8-10, 12-15, 17, 18, 21, 22, 26, 30-32, 35
*BRAF*	7q	NM_004333.4	18	15
*CHEK2*	22q	NM_001005735.1	16	6, 8, 14-16
*CKIT*	4q	NM_000222.2	21	9, 11, 13, 17
*EGFR*	7p	NM_005228.3	28	18-21
*ERBB2*	17q	NM_004448.3	27	19, 20
*FLT3*	13q	NM_004119	24	11, 12, 14, 17
*MYCN*	2p	NM_005378.4	3	2, 3
*NF1*	17q	NM_001042492.2	58	1-58
*NOTCH1*	9q	NM_017617	34	6-8, 11-13, 26, 27, 34
*NRAS*	1p	NM_002524	7	2, 3
*PDGFRA*	4q	NM_006206.4	23	12, 14, 18
*PIK3CA*	3q	NM_006218.2	21	8, 10, 21
*PTEN*	10q	NM_000314.4	9	5-8
*PTPN11*	12q	NM_002834.3	16	3, 7, 8, 12, 13
*RB1*	13q	NM_000321.2	27	1-27
*RET*	10q	NM_020975.4	20	10-16
*SRC*	20q	NM_198291.2	14	12
*STK11*	19p	NM_000455.4	10	1-8
*TIAM1*	21q	NM_003253.2	29	5-29
*TP53*	17p	NM_000546.5	11	5-9

**Table 2:** Technical performance of 106 primary neuroblastoma samples analyzed with the customized NB-Panel

	Mean (Range) for all patients (n=106)
**Mapped reads**	1,623,834 (127,973 – 5,415,928)
**Reads on target**	93.69 % (74.30 – 99.41)
**Mean depth**	2,654 (154 – 9,653)
**Read uniformity**	78.21 % (7.58 – 94.34)
**Mean read length**	147 bp (109-177)

### Distribution of variants in primary neuroblastomas

Sequencing data analysis was performed based on low frequency allelic variants located on exons or splicing sites, except *NF1* gene where both exon and intron low frequency allelic variants were considered. A combination of predictors was used to estimate malignancy. In this manner, the screening for rare variants revealed that only 62 of 106 neuroblastomas (58%) harbor at least one variant in any of the genes included in NB-panel (see Supplementary Table 2 for a detailed description of the identified variants and the predictors used in the determination). The genes most frequently mutated were *ATM* and *NF1* (16 and 15 cases, respectively), followed by *TIAM1* and *ALK* (12 cases each). Most tumors contained no variants or had just one or two, further supporting the simplicity of genetic alterations in primary tumors that gain in complexity later on upon relapse [15]. We also generated an additional list containing the silent variants outside of the splicing sites as well as deep intronic variants beyond 10 bases from the splicing site (Supplementary Table 3). Allelic variants localized 5′ and 3′ UTR were also included in this list. The fact that we failed to detect variants in 42% of the cases suggests that other genetic alterations should be taken into account and therefore, a second version of NB-panel may be required to identify other pathogenic changes in primary neuroblastomas.

### *TIAM1* variant associates with better outcome in primary neuroblastomas

**Figure 1:**
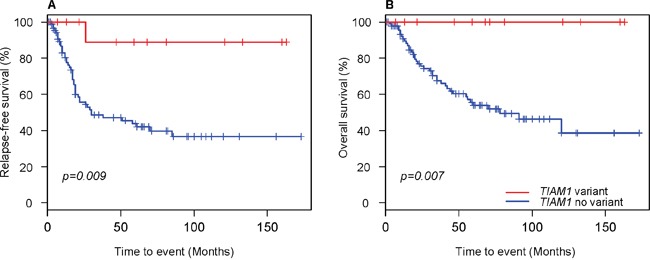
Kaplan–Meier curves for neuroblastoma patients with and without TIAM1 variants **(A)** Relapse-free survival and **(B)** Overall survival. Censored cases are denoted as crosses along the plots. Log-rank p values were used to compare survival curves between patients with and without *TIAM1* variants.

Kaplan-Meier survival analysis revealed that variants in *TIAM1* gene associated significantly with increased disease-free survival (Figure 1A) as well as with overall survival (Figure 1B). All but one of the 12 cases with *TIAM1* variant reported no relapse. Interestingly, this one patient is currently free of disease. No other mutated gene showed a prognostic significance, perhaps owing to low incidence or the need of higher number of cases in the cohort.

**Figure 2:**
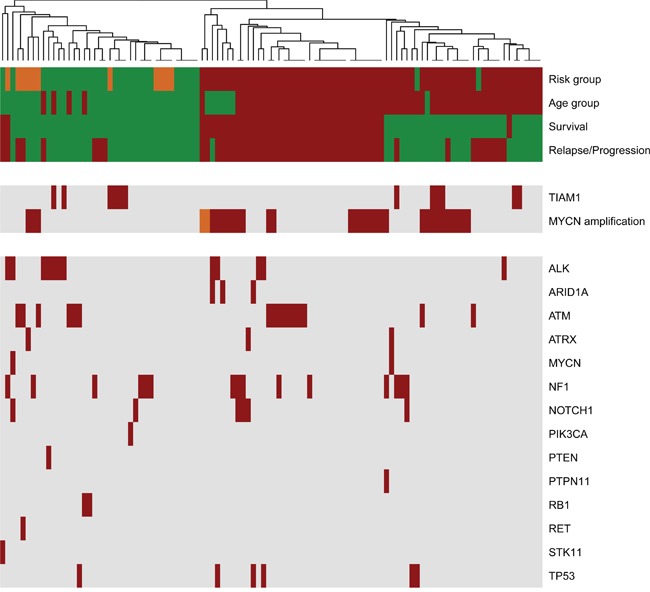
Landscape of clinical variations and identified genetic variants in primary neuroblastomas The heatmap was clustered based on Manhattan distances between cases. Distances were estimated with clinical data and identified variants with NB-panel in 106 primary neuroblastomas and using the Average-Method. Each column summarizes one patient. Each row represents the indicated clinical variable (clustered on the upper rows) or genetic variations (clustered in two groups to highlight those cases with *TIAM1* variants and/or *MYCN* amplification). Risk group (green, low and intermediate risk; red, high risk; orange, stage 4S); Age group (green, <18 months; red, >18 months); Survival (green, alive; red, death), Relapse/Progression (green, disease-free; red, event of progression or relapse); MYCN amplification (green, no amplification; red, amplification; orange, heterogenic amplification); Genes (red, variant found; grey, no variant detected).

Heatmap representation of the clinical parameters and variants revealed that high-risk group clusters together with a defined division between survival and mortality (Figure 2). This distribution suggests that there are genetic and clinical factors in the high-risk group that may predispose for the outcome. *MYCN* amplification is a high-risk factor that can be subdivided into survivors and not survivors within the high-risk group *in the hierarchical* structure of the heatmap (Figure 2). This result also supports the existence of key determinants that define outcome in *MYCN* amplified patients. Among the 11 cases with 4S stage, a metastatic group in children under 12 months of age, 3 had also *MYCN* amplification but did not relapse, further supporting the better outcome in this group [16]. We also identified several variants in 4S stage: 2 cases with a variant in *ATM* gene, 1 case with concomitant *ATM* and *RET* variants, 2 cases in *NF1* and 1 in *ATRX*, all alive and disease free. The only 4S case with a variant in *ALK* underwent progression and was the only one that did not survive, suggesting that ALK inhibitors should also be considered for treatment in 4S stage.

**Figure 3:**
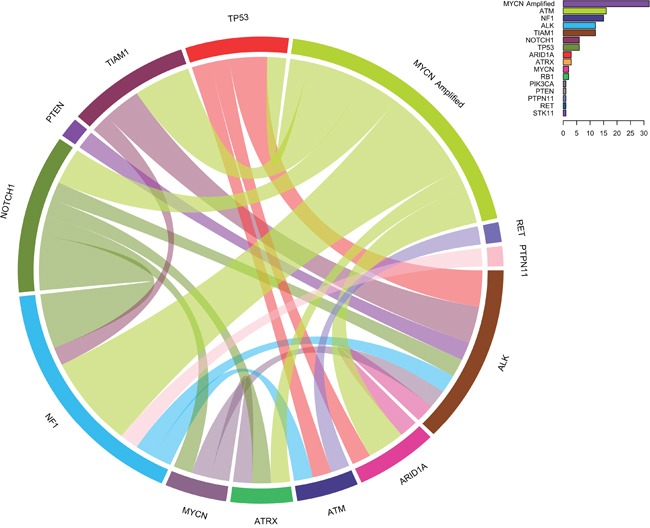
Circos diagram showing concomitantly mutated genes and/or MYCN amplification The diagram represents paired concomitant genetic alterations found in 106 primary neuroblastomas using NB-panel. Frequencies of all variants are indicated in the chart on the upper right corner.

*TIAM1* variants are organized in two groups: the high-risk group and the low/intermediate group (Figure 2). Interestingly, the hierarchical classification for *TIAM1* variants in the high risk clusters in the better outcome subtype (right side cases in Figure 2), suggesting that *TIAM1* variants could be one of the genetic alterations that confer the high-risk group with a better outcome. The coexistence of *TIAM1* variants with other alterations is frequent. In addition to the 3 cases with *MYCN* amplification, 2 cases have concomitant variants/mutations in *ALK* and 1 in *NF1* (Figure 3). Moreover, *TIAM1* variant status is of better prognosis regardless of *MYCN* amplification: out of 32 cases with *MYCN* amplification, 18 died and 14 survived (Table 3). Among the 14 survivors, 3 have a mutation in *TIAM1* gene, one finished treatment and two of them are already free of disease (Table 3). These results strongly suggest that *TIAM1* variant is a genetic alteration that is associated with better clinical outcome, even in the high-risk group with *MYCN* amplification.

**Table 3:** Frequency of *TIAM1* variants in neuroblastomas based on *MYCN* amplification and overall survival

Variables	*MYCN*		Total
	Amplified	Not amp	
		No. with *TIAM1* variant / total	
**Survival**	3/14	9/53	12/67
**Not survival**	0/18	0/21	0/39
**Total**	3/32	9/74	12/106

### *TIAM1* variant is an independent prognostic variable in primary neuroblastomas

To further explore the prognostic value of *TIAM1* variant in neuroblastoma, we performed a multivariate Cox regression analysis. As expected, status of *MYCN* amplification and age were two independent variables that significantly associated with overall survival (Table 4). Noteworthy, *TIAM1* variant was a clear independent variable to predict prognosis in primary neuroblastoma, further supporting its value as an outcome predictor in the disease.

**Table 4:** Multivariate Cox analysis of relapse-free and overall survival for *TIAM1* variant, *MYCN* amplification and age at diagnosis

Variables	Multivariate Analysis					
	Relapse-free survival			Overall survival		
	HR	95% CI	p	HR	95% CI	p
*Age	1.017	1.008-1.025	0.0002	1.010	1.001-1.018	0.022
***TIAM1* variant**						
No	1.000	0.025-0.833	0.021	1.000	0.028-0.206	0.006
Yes	0.222			0.083		
***MYCN* amplification**						
No	1.000	0.723-2.514	0.321	1.000	1.368-5.029	0.004
Yes	1.375			2.623		

Abbreviations: HR, Hazard Ratio; CI, confidence interval

*Age was consider as continuous variable measured in months

### Distribution of variants in *TIAM1* defines an integrated signaling network in primary neuroblastoma

**Figure 4:**
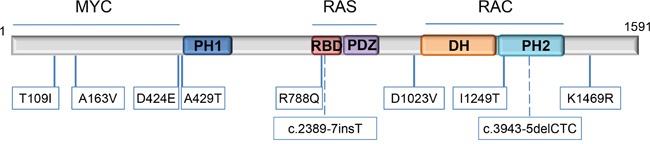
Location of *TIAM1* variants Variants are mapped to specific amino acids on the TIAM1 linearized protein model. The specific domains are indicated (from the N-terminus onwards according to amino acid sequence): PH1: N-terminal PH domain; RBD: RAS-binding domain; PDZ: PSD-95/DlgA/ZO-1 domain; DH: DBL homology domain/RHOGEF domain and PH2: C-terminal PH domain. Locations of the 2 intronic variants are represented by dashed lines. Proteins reported to bind with certain regions of TIAM1 are indicated above the structure.

To understand the role of the identified variants in neuroblastoma etiology and progression, we assessed the predictive effect of each variant in the signaling domains of TIAM1 protein and the clinical features of each patient. As outlined in Figure 4, the variants we identified in *TIAM1* gene are distributed in the three major signaling regions: 1) the N-terminus region that enables the binding to MYC family members to promote transcriptional co-activation and inhibit cellular apoptosis, 2) the RAS-binding domain (RBD) to transduce its signaling to downstream effectors, and 3) the catalytic domain (DH-PH2) that enables RAC activation and thus, activates cellular migration and neuritogenesis. Sequencing of the tumor in parallel with peripheral blood revealed the presence of *TIAM1* germline variants in at least 5 out of the 12 cases (Table 5). No other known pathogenic variants were found in the peripheral blood (Supplementary Table 4). Analysis of paired samples (peripheral blood plus tumor from same patient) revealed no increase of *TIAM1* variant allele ratios in 4 tumors (Supplementary Tables 2 and 4). Notably, 2 tumors showed a *TIAM1* variant allele ratio close to 100% (Supplementary Table 2, variants A163V and D424E), suggesting that they are homozygous in the cancer cells. In one of these patients, the variant was detected in blood cell DNA at a ratio close to 50% (Supplementary Table 4, A163V), supporting a recessive character for this germline variant and that it became the exclusive allele during neuroblastoma formation. These variants in *TIAM1* appear to be rare and improve clinical outcome in neuroblastoma presumably by causing a partial or complete dysfunction of the protein.

**Table 5:** Functional effect predictions and clinical data of patients with *TIAM1* variants

Subject ID	Variant	Germinal	SIFT class	Polyphen-2 class	Mutation taster class	Conclusion	Signaling pathways affected	Age at diagnosis (months)	*MYCN* amplification	Stage (INSS)	Risk group	Relapse / Progression	RFS (months)	Exitus	OS (months)
TI_273	T109I	NA	Damaging	Possibly damaging	Disease causing	Probably reduced protein function	MYCN	24	Yes	3	HR	No	163	No	163
TI_518	T109I	Yes	Damaging	Possibly damaging	Disease causing	Probably reduced protein function	MYCN	5	No	4S	LR	No	68	No	68
TI_464	A163V	Yes	Tolerated	Benign	Disease causing	Possibly reduced protein function	MYCN	1	No	3	LR	No	81	No	81
TI_1036	D424E	NA	Damaging	Probably damaging	Disease causing	Probably reduced protein function	MYCN RAS RAC	47	Yes	4	HR	No	13	No	13
TI_952	A429T	Yes	Damaging	Probably damaging	Disease causing	Probably reduced protein function	MYCN RAS RAC	87	No	4	HR	No	13	No	13
TI_301	R788Q	NA	Tolerated	Probably damaging	Disease causing	Probably reduced protein function	RAS	19	No	1	LR	No	160	No	160
TI_252	c.2389- 7insT	Yes			Polymorphism	Unknown**^1^** Splice site changes	RAS	16	No	4	IR	No	47	No	47
TI_134	D1023V	Yes	Tolerated	Benign	Disease causing	Possibly reduced protein function	RAC	21	No	3	LR	No	133	No	133
TI_384	D1023V	NA	Tolerated	Benign	Disease causing	Possibly reduced protein function	RAC	27	Yes	4	HR	No	121	No	121
NBX_18	I1249T	NA	Tolerated	Possibly damaging	Disease causing	Probably reduced protein function	RAC	44	No	4	HR	Yes	26	No	71
NBX_31	c.3943- 5delCTC	NA			Polymorphism	Unknown**^2^** Splice site changes	RAC	6	No	2	LR	No	22	No	22
TI_480	K1469R	NA	Tolerated	Benign	Polymorphism	Unknown**^3^** Splice site changes	RAC	7	No	4	IR	No	59	No	59

^1^ Splice site changes predicted by NetGene2 and NNSplice; ^2^ Splice site changes predicted by Mutation taster, SpliceView, NetGene2 and NNSplice; ^3^ Splice site changes predicted by Mutation taster and Human Splicing Finder; Abbreviations: HR, High risk; INSS, International Neuroblastoma Staging System; IR, Intermidiate Risk; LR, Low risk; NA, Not available; RFS, Relapse-free survival; OS, Overall survival.

*TIAM1* variants were detected across risk groups, with 5 cases in the low risk, 5 cases in the high risk and 2 cases in intermediate risk (Table 5). *TIAM1* variants are also evenly distributed among the stages, although with a higher tendency towards stage 4. In addition, *TIAM1* variants T109I and D1023V were each found twice, although the distribution was equal in risk groups. These results suggest that *TIAM1* variants do not associate with clinical stages and risk groups and therefore, *TIAM1* variant is an independent factor that improves outcome probably by dysregulating signaling pathways important in neuroblastoma cells.

## DISCUSSION

The implementation of our customized NB-panel in 106 primary neuroblastoma patients confirmed some of previously reported mutated genes in neuroblastoma with very similar frequencies, including *ALK* (11% of the patients) [17–21], *NF1* (14%) [22], *ATM* (15%) [23], and others with lower frequency such as *ATRX* (3%) [20, 21], *ARID1A* (3%) [24] and *STK11* (1%) [25]. Interestingly, we found mutated *TP53* in 6% of the cases, a percentage higher than expected in primary neuroblastomas. Mutations in *TP53* tumor suppressor have been reported to be rare at diagnosis but may be the cause of resistance acquired over chemotherapy [26]. In our cohort, the variant allele ratios for 6 cases with *TP53* variants were found at frequencies close to 100% (2 cases) or close to 50% (4 cases) (Supplementary Table 2). Unfortunately, blood samples are not available to determine if these were germinal or somatic mutations. Nevertheless, it is very improbable that both alleles were inherited from each parent in the 2 cases with homozygous *TP53* mutation. One possibility is that an event of uniparental disomy occurred, leading to a double copy of the inherited *TP53* variant. Alternatively, the normal *TP53* allele may have been deleted or exchanged by the mutated allele, suggesting that although rare, *TP53* homozygous mutations also occur prior to chemotherapy in neuroblastomas. Additionally, we also found one patient with a variant in the receptor tyrosine kinase *RET*, in contrast to a previous report where no *RET* mutations were found in primary neuroblastomas [27]. Although the frequency was very low, our results may support screening for *RET* mutations in precision medicine panels since it is a druggable target that could be use in therapy, alone or in combination with inhibitors of its downstream effectors such as mTOR. Importantly, we noted some patients with concomitant *MYCN* amplification and *NF1* variants (Figure 3) and the survival rate in this group is low (Figure 2). Based on the role of NF1 in RAS/RAF/MEK/ERK signaling, this data suggest that this high-risk group may require concomitant or alternative use of MEK inhibitors to improve outcome.

The role of TIAM1 in the invasion and metastasis of tumor cells has been well documented (reviewed in [28]). However, the oncogenic role of *TIAM1* in neuroblastoma is still not clear. A recent study with 240 high-risk neuroblastoma patients did not identify any mutations in *TIAM1* gene [21]. One plausible explanation is that screening for tumor-specific somatic mutations may have skipped *TIAM1* protective germline variants. In contrast, another study with 87 neuroblastoma patients, investigators reported 3 mutations in *TIAM1* gene, one with concomitant *MYCN* amplification and poor outcome [14]. Our results with 106 primary neuroblastomas reveal that *TIAM1* has a higher incidence of variants (11%), and they significantly associate as an independent variable with better outcome, even in the concomitant presence of *MYCN* amplification or *ALK* mutation. One explanation for the higher percentage of *TIAM1* variants found in our cohort *vs*. previous reports is that we focused strictly on primary neuroblastomas and we took into account both low and high risk groups. We report that *TIAM1* variants improve clinical outcome and are evenly distributed in risk groups (Figure 1 and Figure 2). Hence, restricting the analysis to high risk group or inclusion of relapsed tumors as in published studies may have excluded or reduced the incidence of *TIAM1* mutations. At least 5 out of 12 of these mutations are germline, suggesting that these rare variants could be inherited. Therefore, screening only for somatic mutations by terms of comparing sequences from blood and tumor would have censored these *TIAM1* germline variants. Interestingly, A163V *TIAM1* variant was heterozygous in blood and became homozygous in the tumor, suggesting a recessive phenotype. In addition, variant D424E was homozygous in the tumor and although no DNA from blood was sequenced, ExAC reported this variant in only 10 alleles out of 120256 sequenced, but none of them in homozygous (http://exac.broadinstitute.org/variant/21-32624197-G-T), also supporting a recessive phenotype for this variant. Collectively, our findings suggest that these variants promote a better outcome, presumably by impairing the function of TIAM1. One plausible explanation is that these variants disrupt or induce loss of function in oncogenic signaling networks mediated by TIAM1. All of these variants are localized in the interacting domains that signal to MYCN, RAS, and RAC, pathways that are fundamental for the oncogenic potential of neuroblastoma cells.

T109I and A163V variants are localized within TIAM1 N-terminal domain responsible for the binding to MYC and inhibition of its apoptotic effect [1]. Therefore, these variants may weaken or abolish the binding and facilitate MYC-mediated apoptosis (see Figure 4 and Table 5 for illustrative comprehension). D424E and A429T variants are predicted to inhibit the nearby PH1 domain, highly relevant for TIAM1 subcellular localization [10]. Thus, these variants may dampen or inhibit all TIAM1-dependent signaling pathways. R788Q variant is localized within the RAS binding domain and may inhibit RAS-dependent activation of TIAM1. On the other hand, D1023V is immediately upstream of DH domain, the TIAM1 catalytic domain involved in the nucleotide exchange of RAC. This variant may inhibit RAC signaling by reducing the efficiency of nucleotide exchange. I1249T maps to the PH2 domain which works in tandem with DH to facilitate TIAM1 translocation to the membrane and RAC activation. Therefore, this variant is also predicted to reduce or inhibit RAC signaling.

Turnover of TIAM1 is regulated by the ubiquitin-proteasome pathway: two lysine residues which are sensitive to ubiquitylation (K1404 and K1420) have been localized immediately after the tandem DH-PH2 domain at the C-terminus [29]. K1469 is localized close to the two ubiquitylated lysines and thus, variant K1469R found in one patient may alter TIAM1 localization/turnover or reduce its capacity as a MYCN transcriptional coactivator, as it occurs with other transcriptional coactivators such as NCOA3 and the estrogen receptor [30]. Alternatively, variant K1469R is predicted to generate an alternative splice-site change that may result in reduced mRNA/protein stability (Table 5). The proteasome inhibitor bortezomib with retinoic acid has been suggested as an alternative co-adjuvant therapy in neuroblastoma patients to prevent relapse [31]. Patient with *TIAM1* variant K1469R also harbors an *ALK* variant R1275Q, suggesting that ALK inhibitors could also be an alternative in case of relapse. However, neither this patient nor patient with concomitant *TIAM1* variant R788Q and *ALK* variant F1174L did relapse, further supporting the protective phenotype of *TIAM1* variants in the outcome of the disease. Collectively, *TIAM1* variants associated with better outcome in neuroblastoma patients and therefore, suggest that inhibiting *TIAM1* in neuroblastoma could be used in conjunction with conventional therapy to improve outcome.

## MATERIALS AND METHODS

### Patients and samples

All neuroblastoma samples (106 in total) were derived from primary tumours of untreated patients diagnosed with neuroblastoma between years 1997 and 2015 in Spanish cooperative hospitals. Patients were included in different national and European studies (LNESG I and II, INES, EUNS, LINES, N-AR-99, N-II-92, and HR-NBL1) and carefully selected in order to have all neuroblastoma subtypes represented. Patient's follow-up was 43 months (range 1-173 months). The distribution of patients by stages was 9% stage 1; 7% stage 2; 24% stage 3; 51 stage 4 and 10% stage 4S. Median age of the cohort at diagnosis was 26.35 months (first quartile 10.1 months, third quartile 46.63 months). Median overall survival in the cohort was 43 months (first quartile 16 months, third quartile 76.75 months). *MYCN* was amplified in 30 patients (28%), heterogeneous amplified in 2 patiens (2%) and not amplified in 74 patients (70%). Tumor samples were centrally reviewed and histologic type was classified according to the International Neuroblastoma Pathology Classificarion (INPC) criteria [32, 33]. Staging and risk stratification were established according to International Neuroblastoma Staging System (INSS) and International Neuroblastoma Risk Group (INRG) [34]. Biological studies included status of *MYCN*, 1p and 11q, studied by FISH according to ENQUA guidelines [35, 36]. Patient's follow-up was obtained from clinical records. The study was conducted in accordance with the Declaration of Helsinki and La Fe Research Ethics Committee approved this project. Parents or legal guardians signed an informed consent statement for sample and data management.

Genomic DNA was extracted from frozen tumor tissues by a standard proteinase K and phenol-chloroform extraction protocol. NanoDrop spectrophotometer (Thermo Scientific, Waltham, MA, USA) was used to measure the quality and quantity of the extracted DNA by A260 spectrophotometric absorbance.

### Gene panel design

The customized NB-panel comprises 483 amplicons from 26 genes which are frequently mutated in cancer and/or potential genes to targeted-therapies (see Table 1 and Supplementary Table 1). Design of the panel primers were performed with the Ion Ampliseq Designer (pipeline version 4.2). The panel consists of 2 primer pools; each requires a minimum of 10 ng DNA input material. Panel size is 87.64 kb and amplicon lengths are between 125 and 275 bp.

### Next generation sequencing

Sequencing analysis was performed on the Genomic Unit of La Fe Hospital Research Institute with the Ion PGM™ / Ion PROTON™ system (Life Technologies). Amplicon library was prepared using the Ion Ampliseq Library kit 2.0 (Life Technologies). Briefly, multiplex primer pool was added to 10 ng of genomic DNA and amplified with the following PCR cycles: 99°C for 2 min, followed by 99°C for 15 s and at 60°C for 4 min (17 cycles), and holding on at 10°C. Primers are partially digested using a FuPa reagent, and then sequencing adapters were ligated to the amplicons. The final libraries were purified with Agentcourt AMPure XP (Beckman Coulter) and quantified with Qubit DS HS assay Kit (Life Technologies). DNA template preparation was performed with the semiautomated Ion OneTouch™ 2 instrument and Ion One Touch enrichment system (ES) (Life Technologies). Finally DNA high-throughput sequencing was performed on PGM or Proton instrument. Ion PGM™ HiQ Sequencing kit on the Ion 318 sequencing chip (Life Technologies) was used for PGM instrument. In case of PROTON instrument, we used Ion PI™ HiQ Sequencing Kit on the Ion PI sequencing chip (Life Technologies).

Data from the Ion Torrent runs were analysed using the platform-specific pipeline software Torrent Suite v5.0 for base calling, trim adapter and primer sequences, filtering out poor quality reads and no call reads according to the barcode sequences. The sequence variants in each sample were identified using the Torrent Suite Variant Caller (TSVC; v4.0-r76860) plug-in and browser extensible data (BED) files (chromosome coordinates) that specify the coding regions of the target genes within the human reference genome (hg19) retrieved from the NCBI database (build 37) as a reference. Finally, Ion Reporter software (https://ionreporter.thermofisher.com) was used to further annotate variant caller format (VCF) files and Integrated Genomic Viewer (IGV) software [37] was then used to complete the visualization and to discriminate false-positive variants. We considered only variants with balanced numbers of forward and reverse reads. Based on the American College of Medical Genetics and Genomics and the Association for Molecular Pathology [38], variants with minor allele frequency (MAF) of > 1% in 1000G were considered polymorphisms and were excluded from further analyses. Investigation about the potential pathogenic role was done using databases (COSMIC, dbSNP, ClinVar (NCBI)), prediction algorithms (SIFT, PolyPhen-2 or Mutation Taster) or splice prediction tools (SpliceView, NetGene2, Human Splicing Finder, NNSplice).

### Statistical analysis

Statistics were performed using R software (version 3.2.3). The heatmap diagram was performed to represent the most relevant clinical data and potentially pathogenic variants detected. The distance measure used for heatmap clustering was Manhattan and the clustering method was Average. Circos diagram was generated with R for visualizing data of concomitantly mutated genes and/or *MYCN* amplification. The diagram does not include patients with only one alteration. The survival curves were plotted according to the Kaplan–Meier method and comparisons were performed by log-rank test. A Cox regression analysis was used for multivariate analysis. A p value ≤0.05 was considered statistically significant. Relapse-free survival (RFS) was defined as the time from diagnosis to the time of first occurrence of relapse, progression or last follow-up; and overall survival (OS) was defined as the time from diagnosis until death or until last follow-up if the patient was alive.

## SUPPLEMENTARY TABLES










