# Supplementary material for: TIAM1 variants improve clinical outcome in neuroblastoma
Source: Oncotarget. 2017 Apr 3;8(28):45286–97. doi: 10.18632/oncotarget.16787 (PMC5542186; doi:10.18632/oncotarget.16787)
Supplement: Supplementary file 1 [file oncotarget-08-45286-s001.pdf]

TIAM1 variants improve clinical outcome in neuroblastoma

SUPPLEMENTARY TABLES

Supplementary Table 1: List of the 483 amplicons which are included in the customized NB-panel

See Supplementary File 1

Supplementary Table 2: Potentially pathogenic variants detected with the customized NB-Panel in 106 patients analyzed and functional effect predictions. Selected variants included non-synonymous exonic variants, intronic variants <10 bases to the coding region and *NF1* non-synonymous exonic and intronic variants

See Supplementary File 2

Supplementary Table 3: Other variants detected with the customized NB-panel in 106 patients analyzed: synonymous variants, 3'utr and 5'utr variants and intronic variants (>10 bases to the coding region)

See Supplementary File 3

Supplementary Table 4: Germline *TIAM1* variants detected with the customized NB-Panel in 5 patients with avaiable peripheral blood

See Supplementary File 4
